# Increased M1 Macrophages Infiltration Is Associated with Thrombogenesis in Rheumatic Mitral Stenosis Patients with Atrial Fibrillation

**DOI:** 10.1371/journal.pone.0149910

**Published:** 2016-03-01

**Authors:** Guixin He, Wei Tan, Bingjian Wang, Jianzhou Chen, Guannan Li, Suhui Zhu, Jun Xie, Biao Xu

**Affiliations:** 1 Department of Cardiology, Drum Tower Hospital, Medical School of Nanjing University, Nanjing, China; 2 Department of Cardiology, the First Affiliated Hospitol of Guangxi University of Chinese Medicine, Nanning, China; 3 Department of Cardiology, Huaian First People’s Hospital, Huaian, China; Chang-Gung University, TAIWAN

## Abstract

Atrial fibrillation (AF) is the most common arrhythmia. In patients with AF, the role of macrophage subsets in thrombogenesis is unclear. In the present study, we analyzed the role of M1 and M2 macrophages and related cytokines in thrombogenesis of AF. Immunohistochemistry, Western blot, and TUNEL assay were used to detect M1/M2 macrophage infiltration, the expression pattern of IL-1β and inflammasome components, and apoptosis of cardiomyocytes in 71 specimens obtained from the left atrial appendage of patients with rheumatic mitral stenosis (MS) with or without thrombosis. We demonstrated that proinflammatory M1 macrophages were predominant in the atrium of MS patients with AF and thrombus. NLRP3 inflammasomes and IL-1β, which are primarily functional in macrophages, were activated in those patients. We also showed that increased cell death was associated with thrombogenesis in MS patients. These data indicate that infiltration of M1 macrophages and over-activation of NLRP3 inflammasomes may play a role in progressive atrial inflammation and thrombogenesis in rheumatic mitral stenosis patients with AF.

## Introduction

Atrial fibrillation (AF) is the most common arrhythmia. In addition to deteriorating heart function, AF is associated with a high risk of stroke and systemic embolism and increased mortality, especially in patients with rheumatic mitral stenosis (MS). Thrombogenesis of the left atrium represents the underlying mechanism of AF-related embolism.

Although left atrial appendage velocity is a predominant factor of thromboembolism in AF, inflammation is an independent predictor of thrombogenesis[1]. A variety of inflammatory markers such as C-reactive protein (CRP), tumor necrosis factor-α (TNF-α), interleukin (IL)-2, and IL-6 have been linked to embolism of AF. [2]The underlying mechanisms linking inflammation to thrombosis include production of tissue factor (TF) from mononuclear cells, increased platelet activation and over-expression of fibrinogen. [3–4]

In patients with AF, a pathological study reveals infiltration of macrophages and adjacent myocyte necrosis in atrium, which is absent in patients with sinus rhythm[5]. However, the role of macrophages in thrombogenesis of AF is not clear. Macrophages play a critical role in non-specific defense (innate immunity), and also help initiate and regulate specific defense mechanisms (adaptive immunity) by recruiting other immune cells. Subpopulations of macrophages manifest different proinflammatory or anti-inflammatory responses. Proinflammatory M1 macrophages are recruited early during tissue damage to clear dead cells.[6] By contrast, M2 macrophages, which have reparative functions and secrete proangiogenic or fibrotic mediators to promote wound healing, are recruited after M1 macrophages. No evidence is available supporting quantitative differences between subsets of macrophages or the underlying mechanisms in thrombogenesis of AF.

In the present study, we analyzed the polarization of macrophages and related cytokines in the atrium of patients with rheumatic mitral stenosis with or without thrombus formation to identify the possible role of different subsets of macrophages in thrombogenesis of AF.

## Methods

### Study population and tissue specimens

Consecutive patients with rheumatic mitral stenosis undergoing valve replacement were enrolled between January 2014 and May 2015 at Nanjing Drum Tower Hospital affiliated to Nanjing University Medical School and Huaian First People’s Hospital. The patients were divided into three groups: AF(-)Thrombus(-) group (n = 19), AF(+)Thrombus(-) group (n = 57) and AF(+)Thrombus(+) group (n = 14). Two-dimensional echocardiography was evaluated in each patient pre- and post-operation. Patients with hyperthyreosis, sick sinus syndrome and renal disease were excluded. All medications prior to surgery were continued until the morning of surgery, except warfarin. The operations were as previously described [7].

Left atrial appendage specimens were obtained prior to the establishment of extracorporeal circulation. Part of the tissue was fixed in 4% paraformaldehyde for immunohistochemistry and TUNEL assay. The remaining tissue was frozen in liquid nitrogen and stored at -80°C for Western blot.

All procedures involving human tissue collection and analyses were approved by the Institutional Ethics Committee of Nanjing Drum Tower Hospital (Approval No. 201446) and Huaian First People’s Hospital (Approval No. 201431), and performed in accordance with the principles outlined in the Declaration of Helsinki. All participants enrolled in the present study provided written informed consent.

### Western blot

Tissue samples were washed in phosphate buffered saline (PBS) and homogenized in RIPA solubilization buffer containing a 1% proteinase inhibitor cocktail (Roche) on an ice rotator. The samples were centrifuged at 12,000 rpm for 20 min at 4°C to precipitate the cell debris. The supernatant was transferred into fresh tubes and the protein concentrations were determined by BCA protein assay (Pierce). Equal protein mixtures were electrophoresed in 12% SDS-PAGE and transferred to polyvinylidene difluoride membranes. After blocking with 5% non-fat milk in Tris-buffered saline containing 0.1% Tween 20 (TBST) for 2h at room temperature, the membranes were incubated at 4°C overnight with the following primary antibodies: anti-NLRP3 (1:500, Cell Signaling technology) and anti-Caspase-1 (1:500, Cell Signaling technology). Anti-GAPDH (1:10000, Bioword) was used as the loading control. After washing with TBST, the membranes were incubated with horseradish peroxidase-conjugated goat anti-mouse (1:2000, Bioword) or anti-rabbit antibodies (1:5000, Bioword) at room temperature for 1h. The reactions were developed with enhanced chemiluminescence (Millipore), and images were obtained by film exposure. The bands were quantified using Image J software. All the quantifications of proteins were normalized against GAPDH, and the values of the other two groups normalized by the AF(-)Thrombus(-) group.

### Immunohistochemistry

All tissue specimens were fixed overnight in 4% paraformaldehyde and embedded in paraffin. Paraffin-embedded tissues were cut into 4-μm-thick sections and deparaffinized before endogenous peroxidase quenching and epitope retrieval. After blocking with 1% bovine serum albumin (BSA) in PBS for 30 min, slides were incubated with anti-HLA-DR (Abcam) and anti-CD 163 (Abcam) overnight at 4°C. After washing three times with PBS, slides were incubated with biotinylated secondary antibody for 20 min. Staining was visualized using the VectaStain ABC-AP kit.

### TUNEL assay

To detect apoptosis, TUNEL assay was performed with paraffin-embedded sections using DeadEnd Fluorimetric TUNEL System (Promega), according to the manufacturer’s protocol. Cell nuclei were counterstained with DAPI (Sigma). The number of TUNEL-positive nuclei was manually counted. The total number of nuclei was determined by automatically counting the DAPI using Image J software (NIH, USA).

### Statistical analysis

All continuous variables were described as mean and standard deviation. All categorical variables were described with absolute and relative frequency distributions. Comparisons of means between groups were performed by Kruskal-Wallis test. The χ^2^ test or Fisher’s exact test was used for analysis of differences between groups. Statistical analyses were performed using SPSS version 13.0 software (SPSS Inc. Chicago, Illinois). Statistical significance was defined as *P* value<0.05 (2-tailed).

## Results

### Clinical characteristics

We recruited 90 patients with mitral stenosis undergoing mitral valve replacement surgery. Age, gender, co-morbidities, NYHA classification, serum CRP levels, BNP, BUN, Cr and blood clotting time, were similar in the three groups (Table 1). Although the incidence of stroke or TIA between groups was not significantly different, patients with AF showed a higher risk of embolism (5 in AF + Thrombus—group and 4 in AF + Thrombus + group, vs 0 in AF-Thrombus—group, *P* = 0.09). A slight elevation of INR was noticed in patients with AF (*P* = 0.02). All the patients with AF received warfarin routinely. After enrolling in the Department of Thoracic Surgery, warfarin was stopped and a bridge therapy with LMWH was initiated.

**Table 1:** Clinical data.

	AF(-)Thromb(-)	AF(+)Thromb(-)	AF(+)Thromb(+)	*P* value
	(n = 19)	(n = 57)	(n = 14)	
Age, (years)	49.00±11.22	54.53 ±8.85	55.50 ± 6.88	0.13
Male, (n)	9	16	5	0.30
Hypertension, (n)	2	6	1	0.93
Diabetes mellitus, (n)	1	7	1	0.70
CAD, (n)	0	8	2	0.24
Pulmonary hypertension, (n)	14	49	14	0.09
Congestive heart failure, (n)	1	4	0	0.82
Stroke/TIA, (n)	1	5	4	0.09
Smoke, (n)	2	3	0	0.44
Alcohol abuse, (n)	1	2	0	0.70
Pre-operation data, (n)				
NYHA class I/II/III/IV	1/3/13/2	0/9/44/4	0/1/13/0	0.42
Mitral stenosis	14	42	14	0.10
Mitral regurgitation	19	57	14	NS
Mitral prolapse	0	0	0	NS
Aorta stenosis	5	7	3	0.32
Aorta regurgitation	13	31	5	0.19
Tricuspid regurgitation	16	46	13	0.61
CRP, (mg/L)	3.7±2.61	7.52 ±14.73	9.40 ± 12.82	0.89
BUN, (mmol/L)	5.36±1.52	6.34 ±2.30	6.52 ± 1.84	0.29
Cr, (mmol/L)	65.97±28.74	70.46 ±26.34	73.00 ± 12.34	0.06
BNP, (pg/ml)	112.35±92.71	305.61 ±297.46	307.00 ± 170.73	0.06
PT, (s)	13.64±4.13	15.38 ±5.78	14.35 ± 2.16	0.05
APTT, (s)	49.70±74.95	35.09 ±11.02	39.51 ± 18.80	0.55
INR	1.07±0.25	1.31 ±0.55	1.17 ± 0.23	0.02

### Echocardiography parameters

We compared echocardiography parameters between the three groups pre- and post-operation. In UCG pre-operation, the interventricular septum thickness, left ventricular end-systolic diameter, left ventricular end-diastolic diameter, postero-lateral wall thickness, and ejection fraction were similar in the three groups. Left atrium diameters in patients with AF were significantly larger than those without AF. After operation, left atrium diameters were reduced in all the three groups, compared with pre-operation values (Table 2). In accordance with other studies, left atrium size is the predominant factor contributing to AF [8].

**Table 2:** Echocardiography parameters.

	AF(-)Thromb(-)	AF(+)Thromb(-)	AF(+)Thromb(+)	*P* value
	(n = 19)	(n = 57)	(n = 14)	
Pre-operative				
IVS (mm)	9.32±1.03	8.73±0.83	9.35 ± 0.94	0.07
LVDd (mm)	49.55±9.29	51.41±10.04	47.83 ± 8.59	0.49
LVDs (mm)	34.51±9.64	41.69±9.58	36.90 ± 4.41	0.38
PW (mm)	8.95±0.99	8.65±0.77	9.12 ± 0.92	0.38
LA (mm)	38.57±4.27	51.69±11.30	53.54 ± 7.16	<0.001
EF (%)	56.41±18.08	57.70±7.63	57.62 ± 6.81	0.46
Post-operative				
IVS (mm)	9.58±2.01	8.98±0.90	9.61 ± 1.15	0.21
LVDd (mm)	47.96±7.29	57.35±53.67	47.39 ± 6.18	0.47
LVDs (mm)	36.87±3.71	39.19±7.70	35.87 ± 3.67	0.68
PW (mm)	9.26±1.59	8.84±0.84	9.23 ± 1.09	0.48
LA (mm)	34.27±7.63**	44.53±10.99**	42.46 ± 6.29**	0.001
EF (%)	58.47±8.86	56.69±7.79	58.50 ± 7.00	0.59

IVS: interventricular septum thickness, LVDs: left ventricular end-systolic diameter, LVDd: left ventricular end-diastolic diameter, PW: postero-lateral wall thickness, LA: left atrium diameter, EF: ejection fraction.

**, *P* < 0.01, compared with pre-operative value.

### M1 but not M2 macrophage markers overexpressed in MS patients with AF and thrombus formation

**Fig 1:**
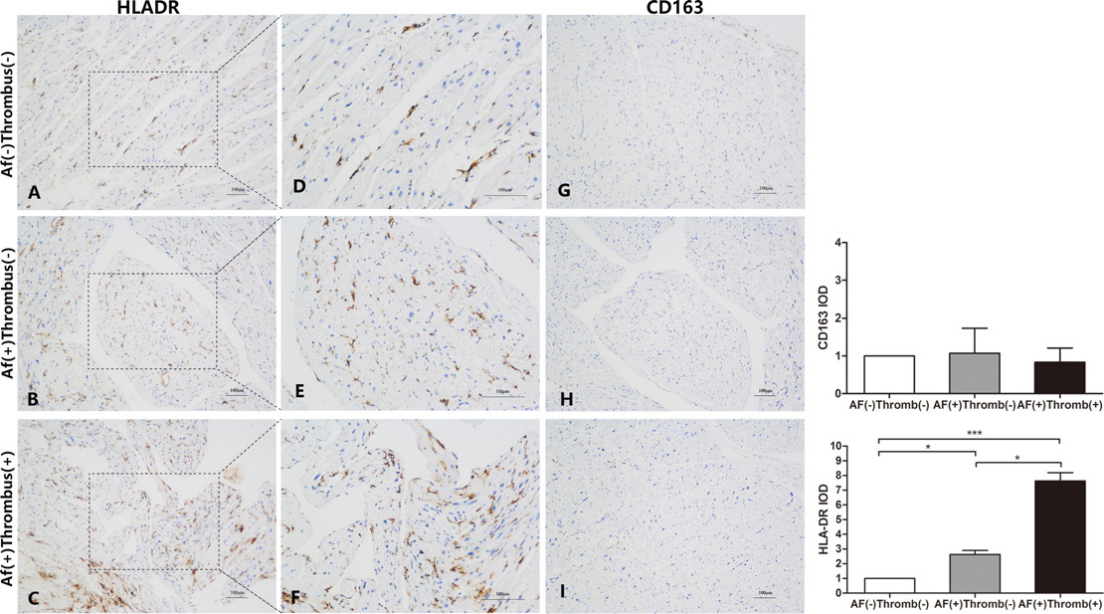
Immunostaining of monoclonal antibodies with anti-HLA-DR for M1 and anti-CD163 for M2 cells. (A-F) M1 cells were predominantly increased and localized in the atrial endothelium of patients with thrombus formation. (G-I) M2 cells were similar in all the three groups.

To investigate whether specific macrophage subpopulations participate in thrombus formation in AF, we first assessed macrophage polarization in the atrium immunohistochemically. HLA-DR was used as a surface marker for M1 macrophages, while CD163 was a marker for M2 macrophages. As shown in Fig 1, there was a clear predominance of M1 over M2 macrophages. HLA-DR was highly expressed in the atrium of MS patients with AF (Fig 1B), when compared with MS patients without AF. HLA-DR was further up-regulated in specimens from AF patients with thrombus formation (Fig 1C). It was noteworthy that several HLA-DR positive cells clustered under the atrial endothelium from AF(+)Thrombus(+) patients, which was not observed in tissue from AF(+)Thrombus(-) and AF(-)Thrombus(-) patients (Fig 1C and 1F). On the other hand, the expression of CD163 was similar among the three groups (Fig 1G, 1H and 1I). These results suggested that excessive M1 macrophage infiltration into atrium may play an important role in thrombogenesis in MS.

### NLRP3 inflammasomes over-activated in MS patients with AF and thrombus formation

We found that excessive M1 macrophages infiltrated into atrium with thrombosis. IL-1β is an important cytokine from macrophages to mediate the inflammatory response. In response to stimuli, the expression of NLRP3 and IL-1β was up-regulated, followed by NLRP3 inflammasome complex with adaptor protein ASC, pro-caspase-1 and maturation of caspase-1[9]. Consequently, mature caspase-1 cleaves pro-IL-1β into its active form IL-1β, mediating cell death[10]. To elucidate whether IL-1β mediates thrombogenic effect of M1 macrophages, we analyzed the expression patterns of NLRP3 inflammasomes and IL-1β.

**Fig 2:**
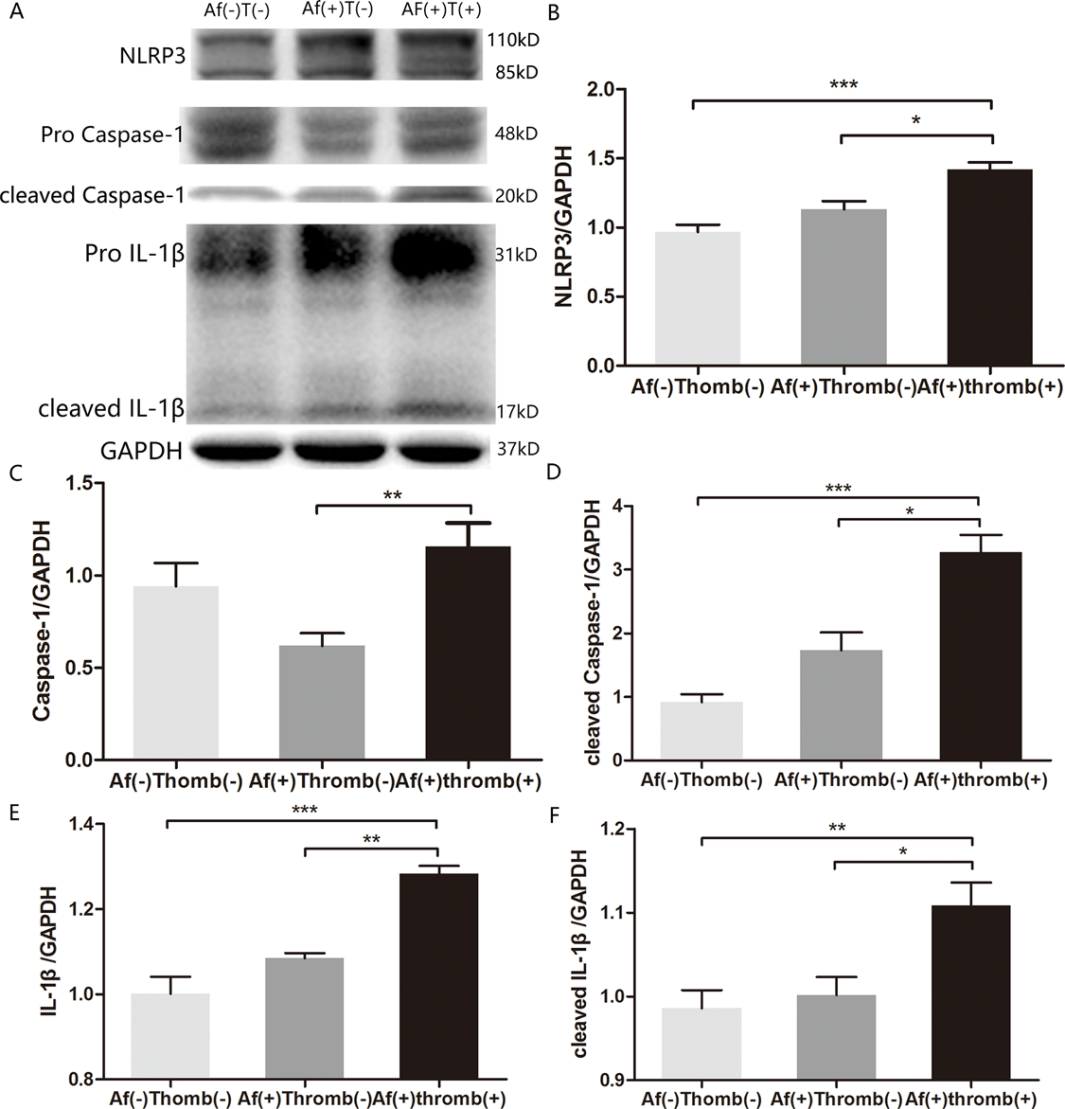
Immunoblot of inflammasome components in lysates of atrium. (A) Representative plot showing expression of NLRP3, pro- and cleaved caspase-1, pro- and cleaved IL-1β. GAPDH represents the loading control. (B-F) Semiquantitative analysis of inflammasome expression; NLRP3, pro- and cleaved caspase-1, and pro- and cleaved IL-1β expressions were significantly increased in patients with AF and thrombosis. * denotes *P* < 0.05, ** denotes *P* < 0.01, *** denotes *P* < 0.0001.

NLRP3 expression was elevated in patients with AF compared with those without AF. In AF, NLRP3 expression was further increased in patients with thrombus formation (Fig 2A and 2B). Although the levels of pro-caspase-1 between AF(-)Thrombus(-) and AF(+)Thrombus(-) group were similar, expression of pro-caspase-1 were significantly elevated in patient with thrombus (Fig 2A and 2C). The levels of cleaved caspase-1, IL-1β and cleaved IL-1β in AF(+)Thrombus(+) patients were significantly higher than those with sinus rhythm, and were elevated when compared with AF(+)Thrombus(-) patients (Fig 2A and 2D–2F). These results showed that in MS patients, NLRP3 inflammasome activation and elevated IL-1β were associated with thrombosis.

### Increased cardiomyocyte death associated with thrombogenesis in MS patients with AF

**Fig 3:**
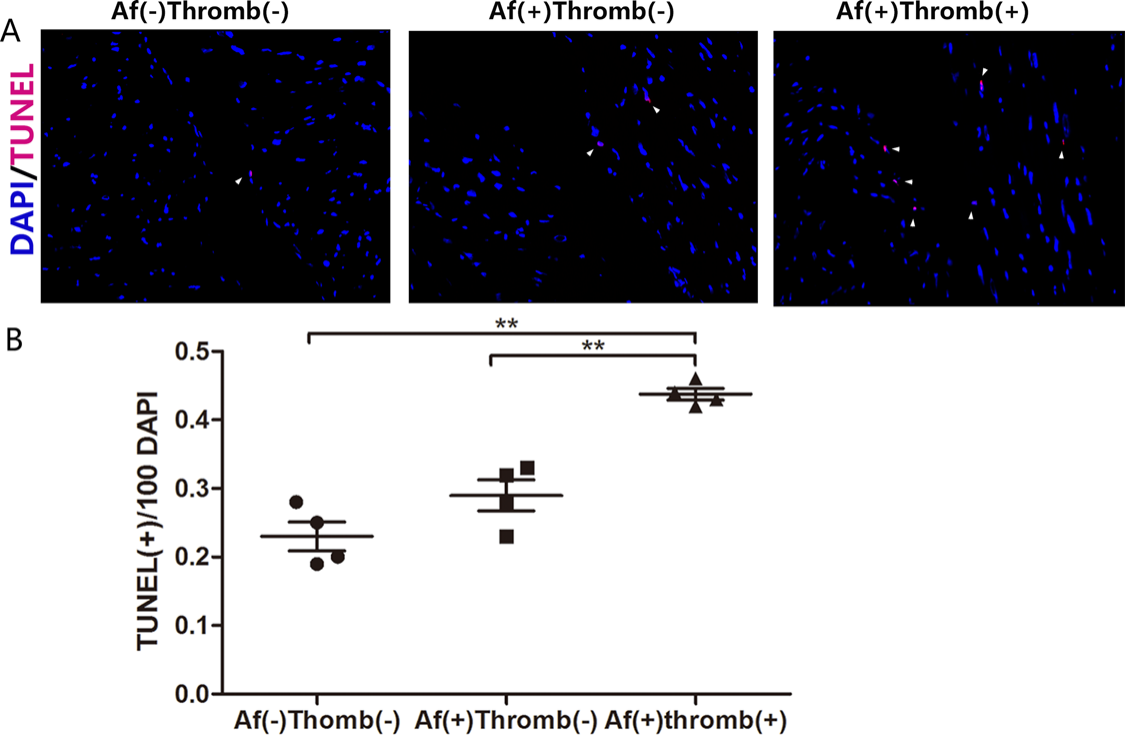
Increased cardiomyocyte death and thrombogenesis in MS patients. (A) TUNEL-positive cells are primarily located in the atrial endothelium. (arrowhead) (B) Frequency of apoptotic cells in the atrium of MS patients as indicated by TUNEL-positive cells per 100 DAPI in three groups. ** denotes *P* < 0.01.

Preceding results indicated that NLRP3 inflammasomes and IL-1β were involved in thrombogenesis in MS patients. The IL-1β is an important mediator of myocyte apoptosis, [11] We used TUNEL assay to investigate apoptosis of additional myocytes in atrium was associated with thrombogenesis. TUNEL-positive cells were significantly increased in AF(+) patient’s atrium. When compared with AF(+)Thrombus(-) patients, the number of TUNEL-positive cells were further increased in MS patients with AF(+)Thrombus(+) (Fig 3, left, middle and right panels).

## Discussion

In the present study, we demonstrated that proinflammatory M1 macrophages were predominant in atrium of MS patients with AF and thrombus. NLRP3 inflammasomes, which are primarily functional in macrophages, were activated in these patients. We also showed that increased cell death was associated with thrombogenesis in MS patients. These data indicate that macrophages play an important role in thrombogenesis of atrial fibrillation.

The role of inflammatory cells in AF and AF-related thrombogenesis is not fully elucidated. Subsets of inflammatory cells show distinct pro- or anti-inflammatory functions. For example, M1 macrophages are pro-inflammatory and clear cell debris during early tissue damage. M2 macrophages have reparative function and secrete fibrotic factors, such as TGF-β, promote tissue healing. They are recruited after M1 macrophages during cell and tissue repair [6]. However, little is known about the role of M1/M2 subsets in the pathogenesis and thrombogenesis of AF. A previous study reported infiltration of lymphomononuclear cells and concomitant necrosis of the adjacent myocytes in the atrium of AF patients, which were absent from the atrium of patients in sinus rhythm [5]. Consistent with this study, we demonstrated massive infiltration of M1, but not M2, macrophages into the atrium of MS patients with thrombus. Additionally, several M1 macrophages were found under atrial endothelium. By contrast, in MS patients without thrombus, relatively fewer number of M1 macrophages were noticed inside the atrium reflecting a local inflammatory process in the atria of MS patients. Early M1 infiltration may represent the initial step of atrial inflammation and massive infiltration into atrial endothelium occurred with progressive inflammation highlighting the pathogenesis of AF-related thrombogenesis.

Although a previous study observed infiltration of macrophage in atrium of AF patients[5], the underlying mechanisms of macrophages in thrombogenesis are still unknown. We postulated that macrophage-endothelial cell injury triggered thrombogenesis in MS patients. Enhanced endothelial expression of VCAM-1 via Toll-like receptor 4 signaling induced atrial thrombogenesis[12].Our TUNEL assay demonstrated that cellular apoptosis was increased in atrial endothelium of patients with thrombus. These data suggested that macrophages induced endothelial cell apoptosis, contributing to thrombogenesis in AF.

IL-1β is an important proinflammatory cytokine that activates innate and adaptive immune responses.[13] The processing of inactive pro-IL-1β depends on NLRP3 inflammasomes. Upon activation via TLRs on macrophages, the transcription factor NF-κB induces NLRP3 and IL-1β gene transcription [14]. NLRP3 and pro-caspase-1 assemble into a complex inflammasome via apoptosis-associated speck-like protein containing a CARD (ASC). This complex activates caspase-1 itself[15], which further cleaves pro-IL-1β into its active form [16]. This process represents post-translational modification of IL-1β. The serum level of IL-1β was altered in AF patients. It mediates myocyte apoptosis[11]. However, its role in AF was not fully known[17–18]. Its role in endothelial cell apoptosis needs further exploration. Immunoblot assay in the present study showed that both pro- and cleaved IL-1β were increased in patients with AF and thrombus. NLRP3 and cleaved caspase-1 expression were significantly elevated in the atrium of MS patients with thrombus strongly indicating that for the first time, and to the best of our knowledge, that NLRP3 inflammasomes were activated and IL-1β was associated with thrombosis in MS patients.

Immunoblot results demonstrated that cleaved caspase-1 and cleaved IL-1β were detected in MS patients with sinus rhythm (Fig 2A).A predominance of M1 marcophages over M2 was seen in the atrium of all patients with rheumatic mitral stenosis. These data suggested that M1 macrophage infiltration and NLRP3 inflammasome activation mediated rheumatic mitral stenosis. In persistent MS, the M1-dependent inflammation was increased, contributing to thrombus formation.

### Limitations

First, all experiments in the present study were performed in human atrial specimens. We could not use gain- or loss-of-function models to demonstrate a causal relationship between over-infiltration of M1 macrophages and NLRP3 inflammasome activation. Second, all patients enrolled were diagnosed with rheumatic mitral stenosis and were undergoing valve replacement. These patients might not represent a majority of AF patients. A further study of non-valvular AF patients is needed to test these hypotheses. Third, although endothelial impairment is a key step in thrombogenesis of AF, we did not counterstain TUNEL with CD31 to identify the endothelial cells.

## Conclusions

Infiltration of M1 macrophages and over-activation of NLRP3 inflammasomes may play a key role in atrial inflammation and thrombogenesis in patients with rheumatic mitral stenosis and AF.

## References

[1] Cianfrocca C, Loricchio ML, Pelliccia F, Pasceri V, Auriti A, Bianconi L, et al C-reactive protein and left atrial appendage velocity are independent determinants of the risk of thrombogenesis in patients with atrial fibrillation. Int J Cardiol. 2010;142:22–28. 10.1016/j.ijcard.2008.12.052 19178964

[2] Patel P, Dokainish H, Tsai P, Lakkis N. Update on the association of inflammation and atrial fibrillation. J Cardiovasc Electrophysiol. 2010;21:1064–1070. 10.1111/j.1540-8167.2010.01774.x 20455973

[3] Kaski JC, Arrebola-Moreno AL. Inflammation and thrombosis in atrial fibrillation. Rev Esp Cardiol 2011;64:551–553. 10.1016/j.recesp.2011.03.015 21616576

[4] Guo Y, Lip GY, Apostolakis S. Inflammation in atrial fibrillation. J Am Coll Cardiol. 2012;60:2263–2270. 10.1016/j.jacc.2012.04.063 23194937

[5] Frustaci A, Chimenti C, Bellocci F, Morgante E, Russo MA, Maseri A. Histological substrate of atrial biopsies in patients with lone atrial fibrillation. Circulation 1997;96:1180–1184. 9286947 10.1161/01.cir.96.4.1180

[6] Chinetti-Gbaguidi G, Colin S, Staels B. Macrophage subsets in atherosclerosis. Nat Rev Cardiol. 2015;12:10–17. 10.1038/nrcardio.2014.173 25367649

[7] Saint LL, Damiano RJ Jr. Surgical treatment of atrial fibrillation. Mo Med. 2012;109: 281–287. 22953591 PMC6179772

[8] Harada M, Van Wagoner DR, Nattel S. Role of inflammation in atrial fibrillation pathophysiology and management. Circ J. 2015;79:495–502. 10.1253/circj.CJ-15-0138 25746525 PMC4457364

[9] Coll RC, Robertson AA, Chae JJ, Higgins SC, Muñoz-Planillo R, Inserra MC, et al A small-molecule inhibitor of the NLRP3 inflammasome for the treatment of inflammatory diseases. Nat Med. 2015;21:248–255. 10.1038/nm.3806 25686105 PMC4392179

[10] Dinarello CA. Interleukin-1 beta, interleukin-18, and the interleukin-1 beta converting enzyme. Ann N Y Acad Sci 1998; 856: 1–11. 9917859 10.1111/j.1749-6632.1998.tb08307.x

[11] Shen Y, Qin J, Bu P. Pathways involved in interleukin-1β-mediated murine cardiomyocyte apoptosis. Tex Heart Inst J. 2015;42:109–116. 10.14503/THIJ-14-4254 25873819 PMC4382874

[12] Katoh S, Honda S, Watanabe T, Suzuki S, Ishino M, Kitahara T, et al Atrial endothelial impairment through Toll-like receptor 4 signaling causes atrial thrombogenesis. Heart Vessels. 2014;29:263–272. 10.1007/s00380-013-0369-3 23754516

[13] Netea MG, Simon A, van de Veerdonk F, Kullberg BJ, Van der Meer JW, Joosten LA. IL-1beta processing in host defense: beyond the inflammasomes. PLoS Pathog. 2010;6:e1000661 10.1371/journal.ppat.1000661 20195505 PMC2829053

[14] Latz E, Xiao TS, Stutz A. Activation and regulation of the inflammasomes. Nat Rev Immunol. 2013;13:397–411. 10.1038/nri3452 23702978 PMC3807999

[15] Lu A, Magupalli VG, Ruan J, Yin Q, Atianand MK, Vos MR, et al Unified polymerization mechanism for the assembly of ASC-dependent inflammasomes. Cell 2014;156:1193–1206. 10.1016/j.cell.2014.02.008 24630722 PMC4000066

[16] Fernandes-Alnemri T, Wu J, Yu JW, Datta P, Miller B, Jankowski W, et al The pyroptosome: a supramolecular assembly of ASC dimers mediating inflammatory cell death via caspase-1 activation. Cell Death Differ. 2007;14:1590–1604. 17599095 10.1038/sj.cdd.4402194 PMC3345951

[17] Parthenakis FI, Patrianakos AP, Skalidis EI, Diakakis GF, Zacharis EA, Chlouverakis G, et al Atrial fibrillation is associated with increased neurohumoral activation and reduced exercise tolerance in patients with non-ischemic dilated cardiomyopathy. Int J Cardiol. 2007;118:206–214. 17027102 10.1016/j.ijcard.2006.03.090

[18] Wang H, Yan HM, Tang MX, Wang ZH, Zhong M, Zhang Y, et al Increased serum levels of microvesicles in nonvalvular atrial fibrillation determinated by ELISA using a specific monoclonal antibody AD-1. Clin Chim Acta. 2010;411:1700–1704. 10.1016/j.cca.2010.07.005 20637189

